# Prognostic Importance and Therapeutic Implications of PAK1, a Drugable Protein Kinase, in Gastroesophageal Junction Adenocarcinoma

**DOI:** 10.1371/journal.pone.0080665

**Published:** 2013-11-13

**Authors:** Zongtai Li, Xiaofang Zou, Liangxi Xie, Hongmei Dong, Yuping Chen, Qing Liu, Xiao Wu, David Zhou, Dongfeng Tan, Hao Zhang

**Affiliations:** 1 Department of Integrative Oncology, Affiliated Cancer Hospital of Shantou University Medical College, Shantou, China; 2 Cancer Research Center, Shantou University Medical College, Shantou, China; 3 Department of Radiation Oncology, Affiliated Cancer Hospital of Shantou University Medical College, Shantou, China; 4 Department of Thoracic Surgery, Affiliated Cancer Hospital of Shantou University Medical College, Shantou, China; 5 Department of Pathology, Affiliated Cancer Hospital of Shantou University Medical College, Shantou, China; 6 Tumor Tissue Bank, Affiliated Cancer Hospital of Shantou University Medical College, Shantou, China; 7 Department of Pathology, University of Rochester Medical Center, Rochester, New York, United States of America; 8 Department of Pathology, The University of Texas MD Anderson Cancer Center, Houston, Texas, United States of America; The University of Hong Kong, Hong Kong

## Abstract

Gastroesophageal junction (GEJ) adenocarcinoma is a lethal cancer with rising incidence, yet the molecular biomarkers that have strong prognostic impact and also hold great therapeutic promise remain elusive. We used a data mining approach and identified the p21 protein-activated kinase 1 (PAK1), an oncogene and drugable protein kinase, to be among the most promising targets for GEJ adenocarcinoma. Immunoblot analysis and data mining demonstrated that PAK1 protein and mRNA were upregulated in cancer tissues compared to the noncancerous tissues. Immunohistochemistry revealed PAK1 overexpression in 72.6% of primary GEJ adenocarcinomas (n = 113). A step-wise increase in PAK1 levels was noted from paired normal epithelium, to atypical hyperplasia and adenocarcinoma. PAK1 overexpression in tumor was associated with lymph node (LN) metastasis (*P*<0.001), advanced tumor stage (*P*<0.001), large tumor size (*P* = 0.006), residual surgical margin (*P* = 0.033), and unfavorable overall survival (*P*<0.001). Multivariate analysis showed PAK1 overexpression is an independent high-risk prognostic predictor (*P*<0.001). Collectively, PAK1 is overexpressed during tumorigenic progression and its upregulation correlates with malignant properties mainly relevant to invasion and metastasis. PAK1 expression could serve as a prognostic predictor that holds therapeutic promise for GEJ adenocarcinoma.

## Introduction

The reported incidence of gastroesophageal junction (GEJ) adenocarcinoma has been increasing remarkably over the past two decades in contrast to the more constant rise of esophageal and progressive decrease of gastric cancers [1]–[3]. Patients with GEJ adenocarcinoma have even poorer survival rates (five-year survival rate, 10%–15%) than do those with other gastric cancers [4]. GEJ adenocarcinoma has been redefined, by Siewert's classification, as a type I to III tumor that crosses the gastroesophageal junction [5]. Increasing evidence suggests that GEJ adenocarcinoma differs from gastric and esophageal adenocarcinomas in molecular signature, pathological evolution, and clinical behavior [6]. The prognosis and therapeutic promise of many molecular factors that have been examined in other cancers (e.g. cancers of the stomach and/or esophagus) are unclear in GEJ adenocarcinoma [7]–[9]. Hence, it is important to find molecular biomarkers of GEJ adenocarcinoma behavior with reliability for predicting outcomes and promise as targets for directed therapy.

In this regard, the biomarker candidates would be measurable not only in terms of their altered level in tumors vs. normal tissues, but contribute to the progression of tumorigenesis. These gene products can be used for diagnosis, outcome prediction, and therapy surveillance and, more importantly, hold promise as direct targets for therapy. Due to the multi-step nature of tumorigenesis, the better candidates should be central to a variety of oncogenic networks activated during tumor progression. In addition, it would be more beneficial if the candidate molecules have been proven to be drugable.

Protein kinases currently constitute a major focus as potential molecular targets and anti-cancer therapeutics [10]–[12]. The p21 protein (Cdc42/Rac)-activated kinase 1 (PAK1) is the founding member of the PAK family (PAK1-6), and the best characterized among the evolutionary conserved family of serine and threonine kinases [11]–[13]. PAK1 has been identified as an effector molecule for the small GTPases Rho, Rac1, and Cdc42 [14], functioning as an integrator and an indispensable node of major growth factor signaling such as epidermal growth factor (EGF) signaling [13], [15]–[20]. PAK1 is overexpressed and hyperactivated in a variety of cancers [13], [21]–[25], and can mediate downstream signaling events that are involved in cytoskeletal reorganization, cell motility, cell cycle, epithelial mesenchymal transition (EMT), invasion and chemoradiotherapy efficiency through multiple layers of mechanisms such as translation, transcription and RNA splicing [13], [26], [27]. The crucial roles of PAK1 in tumorigenesis and metastasis derive from both in vitro and in vivo models and provide the rationale for developing PAK1 inhibitors as anti-cancer agents [10], [11], [16]. Encouraging evidence that many tumors respond to PAK1 inhibitors expands opportunities for the development of novel anti-cancer drugs [10], [11], [16], [28]–[30]. As a recognized oncogene and drugable target in many cancers, including cancers of the gastroinstestinal tract [25], [28], [31]–[33], PAK1 may not only contribute to prognosis, but may also offer new individually tailored therapeutic options for cancers. Yet despite such great promise, PAK1 is mostly unknown in GEJ adenocarcinoma. In this study, we characterized PAK1 expression profiles to investigate its prognostic impacts and therapeutic implications using two cohorts of total 176 samples, and exploiting datasets of tumor samples and cell lines.

## Materials and Methods

### Patients and tissue samples

All patient samples were from the Cancer Hospital of Shantou University Medical College, which is located in the Chaoshan littoral of Southern China and is recognized as a high-incidence region for esophageal cancer in China [34]. We collected 136 formalin-fixed paraffin-embedded specimens from 113 patients with primary GEJ adenocarcinoma undergoing surgery between 2000 and 2002 (median age, 60 years; range, 35–81 years). The specimens were grouped as tumors (n = 113) and surrounding non-cancerous mucosa (n = 23). The patients were followed up for a mean period of 34 months (range, 1–76 months) from the date of surgery. During follow-up, 70 patients (62.0%) died as a result of tumor recurrence or metastasis. Cancer tissue (n = 20) and paired noncancerous samples (n = 20), obtained for immunoblot analysis and immediately snap frozen in liquid nitrogen and kept at −80°C until used, were harvested from another cohort of patients with GEJ adenocarcinomas who underwent surgery at the same institution between November of 2009 and August of 2010.

All tumor samples were identified as type II/III GEJ adenocarcinoma according to Siewert's classification [5]. Here, type II/III GEJ adenocarcinomas were evaluated in accordance with the American Joint Commission on gastric cancer staging system, 7th edition, rather than with the esophageal cancer staging system [35], [36]_ENREF_22. All cases were confirmed by two pathologists. No patients had undergone preoperative radiotherapy or chemotherapy. Signed informed consent was obtained. This study was approved by the Institution Review Board (# 04-070) of the Cancer Hospital.

### Data mining analysis and unsupervised hierarchical clustering

The Oncomine Cancer Microarray database (ONCOMINE; http://www.oncomine.org) was used to study PAK1 mRNA expression in gastric, esophageal, and GEJ adenocarcinoma [37], [38]. Expression PAK1, HER-2 and PCNA values of tumor samples were log-transformed and median centered, and the standard deviation normalized to one per array before comparison to their normal tissue counterparts. Heretofore, unique mRNA expression microarray analysis for pure GEJ adenocarcinoma tissue had been submitted to the Gene Expression Omnibus (GEO; http://www.ncbi.nlm.nih.gov/geo/) with the series accession number GSE22050 [39].

**Figure 1:**
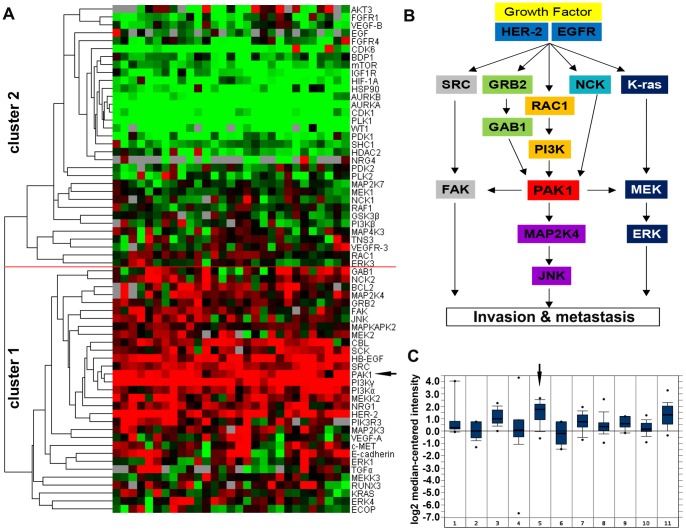
Bioinformatics analysis of PAK1 in GEJ adenocarcinoma. (A) Hierarchical clustering of mRNA expression profiles in the candidate gene set, which comes from the targeted therapy-related signaling of gastric or esophageal tumors [7], [9], based on their similarity to each other in a cohort of 27 GEJ adenocarcinoma cases. Two main clusters were clearly distinguished; overall, expression of genes in cluster 1 was higher than those in cluster 2. HER-2, NRG1, SRC, PI3Ks, PAK1 (arrow), MEKK2 and SCK exhibit prevalently higher mRNA levels than the others. Cluster 1 mainly consists of genes related to HER-2 and EGFR pathways, while cluster 2 consists of genes involving other pathways such as VEGFR and aurora kinase signaling. (B) Putative PAK1-related signaling pathways (modified from KEGG). Cluster color key: red = relative upregulated; green = relative downregulated; black = relative unchanged; grey = missing. (C) A cancer-spectrum analysis of PAK1 expression on ONCOMINE dataset indicated that GEJ adenocarcinoma (arrow) exhibits almost the highest PAK1 mRNA level in all 11 types of cancers. Abbreviations: 1. bladder cancer (n = 8); 2. clear cell renal cell carcinoma (n = 11); 3. colorectal adenocarcinoma (n = 23); 4. ductal breast carcinoma (n = 26); 5. gastroesophageal adenocarcinoma (n = 12); 6. hepatocellular carcinoma (n = 7); 7. lung adenocarcinoma (n = 14); 8. ovarian serous surface papillary Carcinoma (n = 27); 9. pancreatic adenocarcinoma (n = 6); 10. prostate adenocarcinoma (n = 26); 11. squamous cell lung carcinoma (n = 14).

To investigate the similarity of expression patterns, unsupervised hierarchical clustering was performed with Cluster (version 3.0) [40]. The microarray dataset of GEJ adenocarcinomas (GSE22050) was used for cluster analysis. Gene lists in known pathways for targeted therapy were obtained from the Kyoto Encyclopedia of Genes and Genomes (KEGG, http://www.genome.jp/kegg/) database, and were clustered with uncentered correlation, with a similarity metric and average acting as the linkage function. We used Java TreeView (version 1.1) to visualize the clustered data in Figure 1A.

### Immunoblot analysis

PAK1 protein was assayed by immunoblot analysis in human tissue lysates (60 µg of protein in RIPA lysis buffer). Proteins were separated by SDS-PAGE and transferred to a PVDF membrane. The membranes were incubated in blocking buffer (Tris-buffered saline with 0.1% Tween and 5% nonfat dry milk) for one hour and then incubated with rabbit PAK1 antibody (Cell Signaling, Beverly, MA, USA) at a dilution of 1∶1000 in blocking buffer, followed by a horseradish peroxidase-conjugated secondary antibody against rabbit IgG. Signals were visualized with the ECL chemiluminescence system as described by the manufacturer (Amersham Pharmacia, Piscataway, NJ, USA). Blots were reprobed with an anti-actin monoclonal antibody (Abcam, Cambridge, MA, USA) to confirm equal loading of the different samples.

Quantification of the intensity of PAK1 in the Immunoblot was performed by using Bio-Rad Quantity One quantitation software [41], with the ratio between the tumor and the paired nontumor identified as being more than two folds, indicating PAK1 overexpression (see Table S1).

### Immunohistochemistry and staining evaluation

Immunohistochemical (IHC) staining for PAK1, HER-2 and PCNA was carried out using tissue sections (4 µm) that were cut from specimens that had been fixed in 10% buffered formalin and embedded in paraffin. After undergoing deparaffinization, rehydration, endogenous peroxidase blocking, and antigen retrieval (10 mM Tris/1 mM EDTA, pH 9.0, microwave treated), specimens were incubated with a rabbit polyclonal antibody against human PAK1 (1∶100; Cell Signaling, Beverly, MA, USA) or HER-2 (1∶50; Santa Cruz, CA, USA), and a mouse monoclonal antibody against human PCNA (1∶400; PC10, BioGenex, USA) overnight at 4°C. PAK1 cytoplasmic staining, HER-2 membrane staining and PCNA nuclear staining were evaluated. Nuclei were counterstained with hematoxylin. Negative controls were composed of identically treated histological sections with rabbit or mouse IgG to replace primary antibodies.

The staining evaluation and selection of the cut-off point score were performed as follows: ten random 400× microscopic fields per slide were evaluated by two independent observers who were blinded to the clinical information. PAK1 staining was assessed using the semiquantitative histological score (H-score) approach [42], which combines the intensity and number of positive cells. The mean percentage of positively stained cells was scored as follows: 0% (0); 1%–25% (1); 26%–50% (2); 51%–75% (3); and 76%–100% (4). Staining intensity was categorized as follows: absent (0); weak (1); moderate (2); and strong (3). A final score for each specimen was computed using the formula: H-score  =  proportion score × intensity score. Receiver operating characteristic (ROC) curves were used to assess the potential for using the H-score in triage scenarios to detect the clinicopathological characteristics and outcome of GEJ adenocarcinoma. The cut-off score for PAK1 overexpression was the threshold with the objectively best sensitivity and specificity. HER-2 IHC expression was scored as follows: 0 (no staining or faint membrane staining), 1+ (faint membrane staining in >10% of tumor cells, incomplete membrane staining), 2+ (weak to moderate membrane staining in >10% of tumor cells), and 3+ (intense circumferential membrane staining in >10% of tumor cells). PCNA index was determined by the percentage of the cells positively stained by PCNA in the nucleus.

### Statistical Analysis

Statistical analyses were performed using the Statistical Package for Social Science version 17.0 (SPSS, Inc., Chicago, IL, USA) and GraphPad Prism version 5.02 (GraphPad Prism, Inc. San Diego, CA, USA). Simple linear regression analysis was used to test the association of PAK1 expression with HER-2 or PCNA expression. A one-way analysis of variance was used for comparisons with multiple variant groups. Immunohistochemical performance was assessed using a ROC analysis. Areas under the ROC curves (AUC) and 95% confidence intervals (CIs) were estimated to assess differences [43]. Correlations between PAK1 expression and clinicopathological features were analyzed using the Pearson X^2^ test of association or Fisher's exact test. Overall survival rates were generated using the Kaplan-Meier method and survival curves were compared with the log-rank test. Significant factors were identified by univariate analysis, and further examined by multivariate regression analysis with the Cox hazards model. A *P* value <0.05 defined statistical significance.

## Results

### Potential of PAK1 as Molecular Target for GEJ Adenocarcinoma

The initial step of the study was to search potential targets for GEJ adenocarcinoma by means of bioinformatics analysis. First, we scanned the GEO database for suitable datasets of well-defined GEJ adenocarcinomas. This search identified only one independent mRNA dataset comprised of 27 cases of pure GEJ adenocarcinomas (GSE22050) [39]. We next generated a set of 64 potential therapeutic target genes, which have been reported to have therapeutic potential in either esophageal or gastric adenocarcinoma, or both [7], [9], since there is little information of related targets specifically focusing on GEJ adenocarcinoma. Hierarchical clustering of this GEO dataset was performed with the set of 64 genes relating to therapeutic potential, which allowed us to find patterns from all of the genes analyzed. The analysis yielded two differentially expressed clusters: a highly expressed and a low (cluster 1 and 2 in Figure 1A). PAK1 was among the most highly expressed genes in cluster 1 (Figure 1A, arrow) and appeared to be highly related with HER-2, PI3K, SRC, MAP2K4, MEK2 and NCK (cluster 1 in Figure 1A), all of which are known to be components of EGFR and HER-2 signaling pathways [31], [44]–[49] (Figure 1B). Both EGFR and HER-2 pathways are reported to be important to GEJ tumor formation [7], [8], [50], while PAK1 is considered to be the node integrally tied to these growth factor pathways (Figure 1B) [18], [20], [51]. In line with this hypothesis, analysis of the silicon data set identified a close correlation between PAK1 and HER-2 transcripts in GEJ adenocarcinoma (Figure S1), and this was further validated by IHC analysis in surgical specimens of GEJ adenocarcinoma (n = 46) (Figure S1). Since PAK1 is a well-known oncogenic protein in many types of cancers, we weighed the importance of PAK1 in GEJ adenocarcinoma against other cancers. Interestingly, cancer-spectrum analysis of PAK1 expression derived from an ONCOMINE dataset indicated that GEJ adenocarcinoma exhibits one of the highest PAK1 mRNA level in all 11 types of cancers (Figure 1C, arrow). These data suggest that PAK1 might be a potential target for GEJ adenocarcinoma important in the network involved in GEJ tumorigenesis.

### Protein and RNA levels of PAK1 in Cancer and Noncancerous Tissues

**Figure 2:**
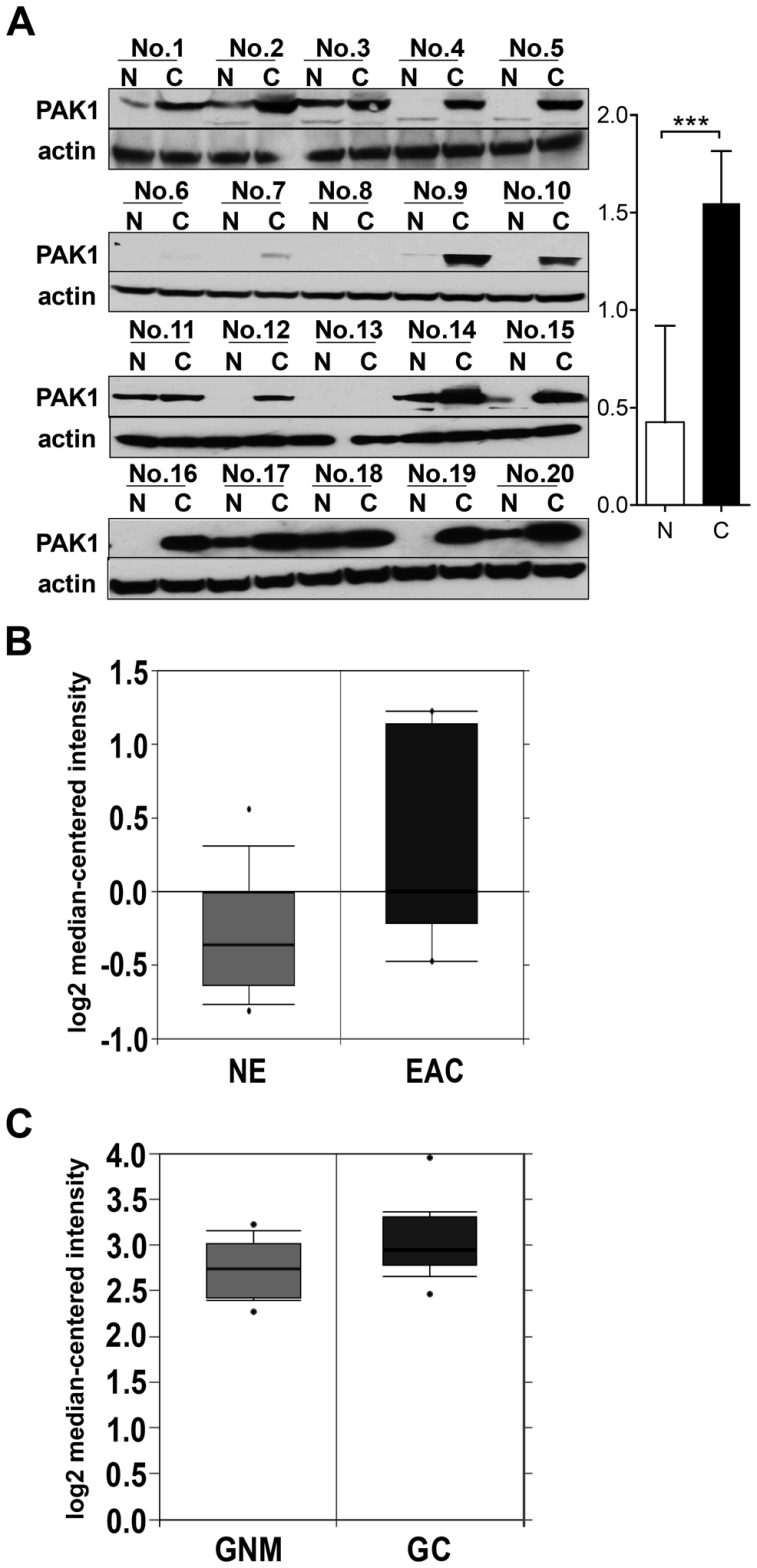
Upregulated PAK1 in GEJ adenocarcinoma. (A) PAK1 protein levels were determined by immunoblot analysis in human GEJ adenocarcinoma tissues (C; n = 20) vs. adjacent noncancerous tissues (N; n = 20); ***P<0.001. PAK1 mRNA levels in esophageal adenocarcinoma (B) and gastric cancer (C) were analyzed using the ONCOMINE database (http://www.oncomine.com). NGM, normal gastric mucosa (n = 12); GC, gastric cancer (n = 12); NE, normal esophagus (n = 24); EAC, esophageal adenocarcinoma (n = 9).

Encouraged by the above data that suggest PAK1 is a molecular target for GEJ adenocarcinoma, we were interested in examining if PAK1 was altered at both protein and RNA levels in tumors vs. normal tissues. A cohort of GEJ adenocarcinomas (n = 20) assayed by immunoblot analysis demonstrated that 75% of GEJ adenocarcinoma tissues (15/20) showed higher PAK1 expression than in the adjacent noncancerous tissues (Figure 2A, *P*<0.001). Of note is that the remaining 25% (5/20) of tumor samples did not exhibit noticeably upregulated PAK1 versus paired noncancerous controls, of which the majority (3 out of 5) were at the early TNM stage (Table S1). Furthermore these samples lacked evidence of lymph node (LN) metastasis, implying an impact of PAK1 on metastatic properties in GEJ adenocarcinoma (also see below and discussion). To support the above observation, we then conducted data mining analysis on ONCOMINE (http://www.oncomine.org). Since there is no array data available for GEJ adenocarcinoma with paired noncancerous tissues, we mined data from gastric cancer and esophageal adenocarcinomas, both of which are closely relevant to GEJ adenocarcinomas. Both in stomach and esophagus, PAK1 transcripts were higher in cancer tissue than in normal tissue (both *P*<0.05, Figure 2B and C). Altogether, these findings clearly indicate that PAK1 is upregulated in tumor tissues compared to normal controls, in agreement with the oncogenic potential of PAK1 in GEJ adenocarcinoma.

### PAK1 Expression during Tumor Advancement

**Figure 3:**
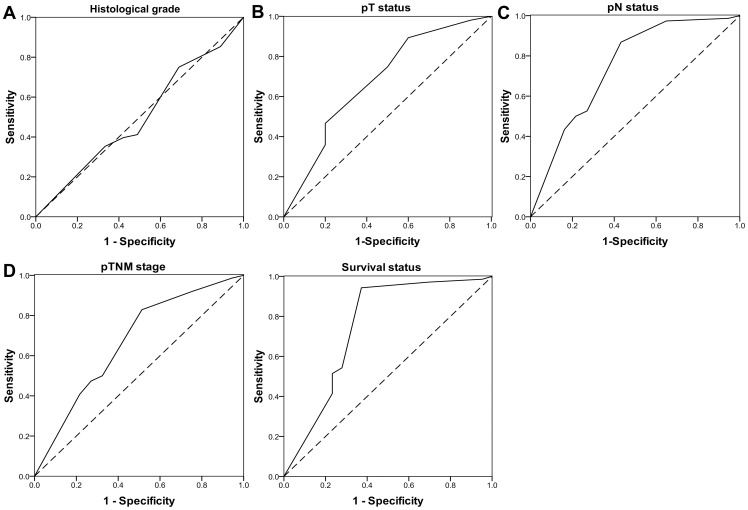
ROC analysis for the immunohistochemistry cut-off score. The sensitivity and specificity for each clinical outcome were plotted (A–F). Accuracy was measured by the area under the ROC curve (AUC). The dashed line indicates a reference test threshold with an AUC of 0.5. The AUC > 0.5 with a statistical significance represents a worthy test. pT status, depth of tumors; pN status, LN status.

We further investigated the expression profile of PAK1 in GEJ adenocarcinoma by utilizing immunohistochemistry. To develop a reasonable cut-off score for PAK1 overexpression, ROC curve analysis was performed. The cut-off score for PAK1 overexpression was 6 (H-score), which was closest to the point with maximum sensitivity and specificity (Figure 3). The cases with scores lower than or equal to the cut-off value were designated as having normal expression, whereas those with higher scores had overexpression.

**Figure 4:**
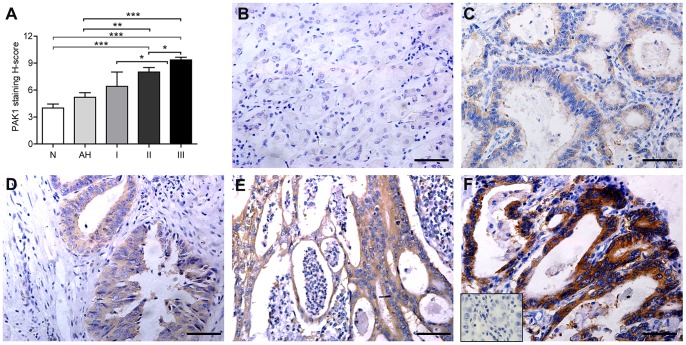
H-score and representative immunohistochemical stainings. (A) The H-score of surrounding normal GEJ epithelium (N, n = 9), atypical hyperplasia (AH, n = 14) and stage I–III GEJ tumor (n = 6, 31, and 76, respectively); error bars represent “mean + SEM”; ***P<0.001, **P<0.01, *P<0.05. (B) Surrounding normal GEJ epithelium is not or weakly stained. (C) An atypical hyperplasia exhibits piling up of diffused and light staining. (D) Light-to-moderate diffused staining in a stage I GEJ tumor. (E) Diffused and moderate staining in a tumor in stage II GEJ tumor. (F) A GEJ tumor in stage III is strongly stained. Insert in (F) showed rabbit IgG as negative control. Scale bar  =  50 µm.

PAK1 overexpression was observed in the cytoplasm of neoplastic cells in 72.6% of primary tumors (n = 113). A step-wise elevation of PAK1 H-score (mean±SEM) in tumor progression: surrounding normal GEJ epithelium (4.000±0.441), atypical hyperplasia (5.200±0.509), stage I tumor (6.400±1.600), stage II tumor (8.000±0.505), and stage III tumor (9.360±0.296) (Figure 4). Thus, these observations clearly suggested that PAK1 expression is enhanced with tumor advancement in GEJ adenocarcinoma.

### Correlation of PAK1 Expression with Clinicopathological Characteristics

We then examined the relationship of PAK1 expression with the clinicopathological features in GEJ adenocarcinoma. PAK1 overexpression was associated with LN metastasis (*P*<0.001) (Table 1). pTNM stage III patients had higher PAK1 expression than did stage I and II patients (*P*<0.001). In addition, high expression was associated with large tumor size (>6 cm vs. ≤6 cm, *P* = 0.006) and positive residual surgical margin (*P* = 0.033), while PAK1 levels were not remarkably altered between tumors of different histological grades (Table1; Figure S2). Given that PAK1 has been reported to regulate cell proliferation in several cancers [21]–[25], we further examined if PAK1 is correlated with PCNA, a cell proliferation marker, in GEJ adenocarcinoma. Both IHC assay and analysis of silicon data set did not show a close correlation between PAK1 and PCNA (Figure S3). Together, these data demonstrate that PAK1 overexpression is associated with malignant properties mainly relevant to invasion and LN metastasis and thus support the hypothesis that the altered PAK1 may contribute to tumor progression of GEJ adenocarcinoma.

**Table 1:** The Clinicopathological Characteristics Related to PAK1 Expression in GEJ Adenocarcinoma.

Parameters	No. of patients	Normal expression	Overexpression	*P*-value
Age				
≤60 years	61	21	40	0.072
>60 years	52	10	42	
Gender				
Male	92	27	65	0.342
Female	21	4	17	
Tumor Size				
≤6 cm	72	26	46	0.006
>6 cm	41	5	36	
Depth of Tumors				
pT1–pT2	10	5	5	0.095
pT3–pT4	103	26	77	
LN Status				
Negative	37	21	16	<0.001
Positive	76	10	66	
pTNM Stage				
I–II	37	18	19	<0.001
III	76	13	63	
Histological Grade				
1–2	45	14	31	0.478
3	68	17	51	
Resection Margin				
Complete	102	31	71	0.033
Residual	11	0	11	

### Association between PAK1 Expression and Patient Survival

**Figure 5:**
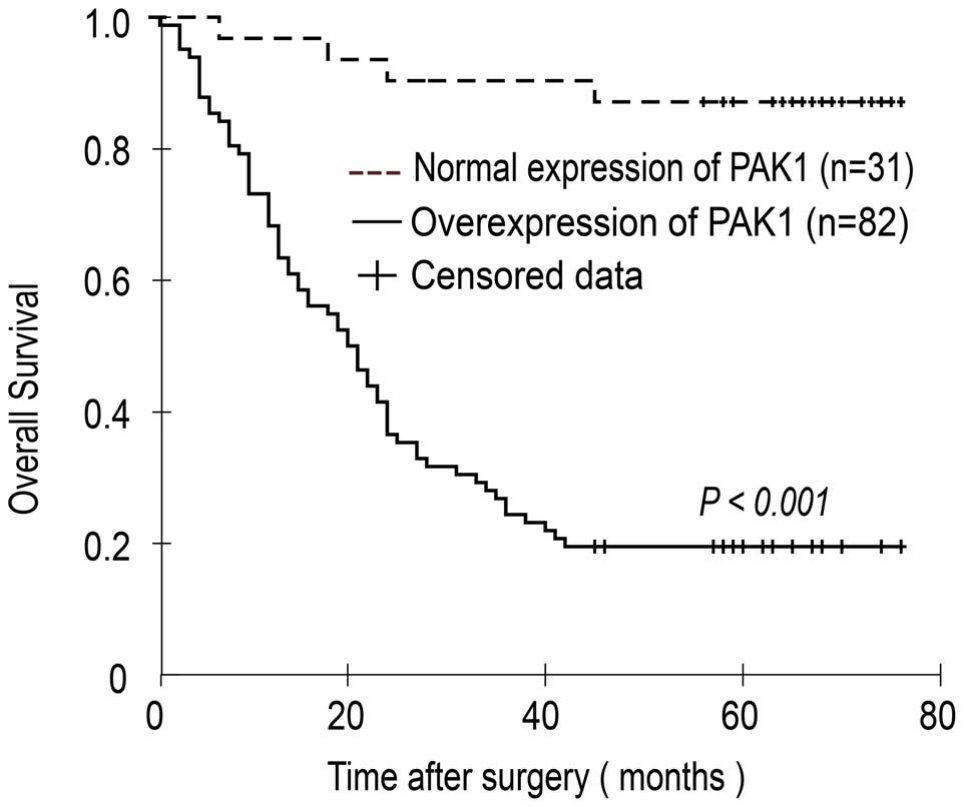
Relationship between overall survival and PAK1 expression. Kaplan-Meier analysis shows PAK1 overexpression was significantly associated with shorter overall survival for patients with GEJ adenocarcinoma.

In order to assess the prognostic impacts of PAK1 expression on the outcome of GEJ adenocarcinoma patients, Kaplan-Meier survival analyses were performed. As shown in Figure 5, the overall survival time (mean ± SD) of patients with PAK1 overexpression was less than half of those with normal expression (31.335±2.703 months vs. 69.484±3.133 months, *P*<0.001, log-rank test). Accordingly, low expression of PAK1 is strongly associated with prolonged survival of GEJ adenocarcinoma patients.

### Univariate and Multivariate analysis

PAK1 expression was related to overall survival following resection (Table 2). On univariate analysis, PAK1 expression, sex, age, pTNM stage, LN status, histological grade and tumor size were responsible for outcomes of patients who underwent surgical resection (Table 2). We subsequently determined the value of PAK1 expression as an independent prognostic predictor. After adjusting the prognostic factors which were established in univariate analysis, only PAK1 expression, pTNM stage, histological grade, tumor size, and sex maintained independent significance for overall survival (*P*<0.001, *P* = 0.045, *P* = 0.031, *P*<0.022 and *P*<0.010, respectively; Table 2). Results revealed PAK1 overexpression as an independent high-risk predictive indicator for the prognosis of GEJ adenocarcinoma patients.

**Table 2:** Univariate and Multivariate Cox Proportional Hazards Model Predicting Survival in GEJ Adenocarcinoma.

Parameters	Univariate analysis		Multivariate analysis	
	RR (95% CI)	*P*-value	RR (95% CI)	*P*-value
Gender				
Female vs. Male	2.038 (1.188−3.496)	0.01	2.063 (1.192−3.572)	0.01
Age (years)				
>60 vs. ≤60	1.786 (1.114−2.863)	0.016		
Depth of Tumors				
T3–T4 vs. T1–T2	2.840 (0.892−9.040)	0.77		
Histological Grade				
3 vs. (1–2)	1.737 (1.053−2.867)	0.031	1.741 (1.051−2.886)	0.031
LN Status				
pN1-3 vs. pN0	3.061 (1.671−5.607)	<0.001		
Tumor Resection Margin				
Complete vs.Residual	1.931 (0.980−3.805)	0.057		
PAK1 Expression (#)	11.522 (4.187−31.871)	<0.001	10.872 (3.915−30.192)	<0.001
Adjuvant Chemotherapy				
Yes vs. No	0.700 (0.409−1.197)	0.192		
Tumor Size				
>6 cm vs. ≤6 cm	2.126 (1.325−3.413)	0.002	1.751 (1.082−2.833)	0.022
pTNM Stage				
III vs. (I–II)	2.544 (1.434−4.513)	0.001	1.824 (1.014−3.283)	0.045

RR, relative risk; CI, confidence interval; (#), overexpression vs. normal expression.

## Discussion

Here we employed multiple approaches to provide the first comprehensive analysis of PAK1 in a large series of 176 samples from patients with primary resected GEJ adenocarcinoma and the relevant microarray datasets. We found that PAK1 is highly upregulated in tumor tissues vs. the noncancerous tissues. PAK1 expression correlated with malignant properties and was an independent prognostic indicator. Our data also support the notion that PAK1 is an important node in the PAK1/HER-2/EGFR network and may be a targetable molecule for GEJ adenocarcinoma. Thus, our study underscores the importance of PAK1 as a prognostic biomarker that also holds great therapeutic promise for GEJ adenocarcinoma.

Thus far, our understandings of cancer biomarkers and therapeutic targets for GEJ adenocarcinoma have lagged behind those of gastric and esophageal cancers. The main reasons for this include unavailability or difficulty in obtaining model systems, such as proper cell lines and gene-engineered animals. On the other hand, it is difficult to draw clear conclusions due to the majority of previous gastroesophageal tumor sample studies having already been mixed by tumor heterogeneity (squamous carcinoma vs. adenocarcinoma) [52], multiple tumor sites (esophagus, gastroesophageal junction and stomach) [53]–[55], and perhaps consequent involvement of clinical behavior. Moreover, gene expression microarrays discovered important prognostic information and suggested new pathways. However, microarray research of GEJ adenocarcinoma remains very limited. Thus, well-defined resected specimen of patients and proper gene-expression datasets from GEO (GSE22050) are currently applicable avenues for studies of GEJ adenocarcinoma. In this regard, this is the first study to explore molecular targets and biomarkers by means of combined these two avenues.

Although an immense number of proteins have been assessed for their prognostic value and therapeutic potential in gastric and esophageal cancers, the effective biomarkers, particularly those with great therapeutic promise, for GEJ adenocarcinoma are few. Our screening using the unsupervised hierarchical cluster analysis suggests that PAK1 is a leading candidate among all reported possible target candidates. PAK1 is integrally tied to the HER-2 and EGFR networks, although it is unknown if PAK1 is directly regulated by HER-2 or EGFR [19], [56]. Consistently, our clustering results suggest that PAK1 is closely related with components of the HER-2 and EGFR networks. Currently, HER-2 and EGFR are few known molecules whose therapeutic effects for GEJ adenocarcinoma have been established in clinical trial [7], [8], [50]. In contrast to the failure to improve outcome by the majority of targeted agents in gastric cancers, a phase III trial demonstrated that trastuzumab, a HER-2 monoclonal antibody, substantially enhanced the response rate and overall survival of a group of patients with HER-2 overexpressing gastric cancer, including patients with GEJ adenocarcinoma [50], [57]. In line with this finding, our IHC assay in patient samples (n = 46), validated by data mining analysis, indicates that PAK1 is positively associated with HER-2 expression in GEJ adenocarcinoma. Previous study has provided supportive evidence that combined targeting PAK1 with current target therapeutic drugs may enhance anticancer effects [28]. Thus, therapeutic strategy targeting PAK1/HER-2/EGFR network holds promise for the treatment of GEJ adenocarcinoma. Our cancer-spectrum analysis reveals that PAK1 expression in GEJ adenocarcinoma is higher than in most other forms of tumors, suggesting that PAK1 overexpression is selective for this type of cancer. Since PAK1 is an oncogene and drugable protein kinase, targeting PAK1 may represent an avenue for improvement of GEJ adenocarcinoma therapy.

Although PAK1 has been studied in gastrointestinal cancers, the role of PAK1 in GEJ adenocarcinoma remains missing [21], [24], [25], [31], [33], [58]. Our analysis using IHC, immunoblot analysis, and data mining indicates that PAK1 is highly differentially over-expressed in GEJ tumor tissues compared with noncancerous tissues. Similarly to our study, PAK1 protein overexpression and copy number gain were found in more than 40% of gastric cancer tissues [25], [59], and advanced gastric cancer tissues express higher PAK1 levels than do matched noncancerous adjacent mucosa [58]. PAK1 was also reported to be amplified in circulating esophageal squamous cell carcinoma cells [60]. Moreover, PAK1 expression levels are significantly lower in normal colon tissue than in colorectal adenomas and invasive neoplasms [21]. As is not reported previously, we noticed an enhanced expression of PAK1 from adjacent nontumorous tissues and atypical hyperplasia to various degrees of tumors from stage I to III, suggesting a selection for increased PAK1 expression during tumor progression. These data underlie the concept that PAK1 plays a vital role in tumorigenesis and progression of GEJ adenocarcinoma.

Our analysis documents that a connection exists between PAK1 expression with the advanced tumor stage, tumor size (> 6 cm), LN metastasis, and resection status. Our data are supported by the report that PAK1 is correlated with LN metastasis, lymphatic invasion, tumor size and tumor stage ([III+IV] > [I+II]) in gastric cancer [58]. Data derived from both IHC of specimens and analysis of silicon data set did not show an association between PAK1 and PCNA, a cell proliferation marker, in GEJ adenocarcinoma. Besides, western blot assay of specimens also showed that noticeable upregulated PAK1 in tumors versus paired nontumors were mainly observed in those with LN metastasis or high TNMs staging. Similarly, PAK1 expression in colorectal tumor tissue is associated with LN status, distant metastasis and tumor stages [24]. PAK1 is known to promote invasiveness of cancer [21]–[25]. However, we did not discover a significant correlation between PAK1 overexpression and invasion depth in current study, which is disagreeable with findings studied in gastric and colorectal cancers [24], [25]. One of possibilities may be due to the actual composition of samples in which 91.2% (103/113) were T3–4 samples, while T1–2 samples were barely 8.8% (10/113). Careful examination of sections revealed that stronger PAK1 expression was in the samples with more severe cancer-cell infiltration away from main tumor mass breaking into surrounding tissues, as compared with those with less severe cancer-cell infiltration (Figure S4). Further study using larger samples with T1–2 staging tumors may be more informative to address the question. Overall, our results suggest that PAK1 expression is closely associated with tumor invasion and LN metastasis rather than cell proliferation in GEJ adenocarcinoma.

According to our Kaplan-Meier estimate, PAK1 overexpression in GEJ adenocarcinoma was correlated with shorter overall survival, which is agreed by previous studies that PAK1 overexpression is correlated with a decreased overall survival in gastric, colorectal, hepatocellular and breast cancer patients [21], [61]–[63]. However, another study reported that the PAK1 low-expression group had a unfavorable prognosis among gastric cancer patients [25]. The discrepancy may reflect the distinctive cell contexts of different forms of tumors, or alternatively may also arise from several sources, e.g., tumor grade/stage, differential patient treatment, antibody efficiency, scoring method, and other methodological aspects related to the IHC.

In this study, the GEJ adenocarcinoma samples were obtained from a littoral in China with a high-incidence of esophageal cancer [64], [65]. All tumors were defined as type II and III tumors according to Siewert's classification [5]. Thus the significance of PAK1 in GEJ adenocarcinoma in this study may be largely confined to the type II and III, which are more prevalent in Asian populations [66]. It remains to be investigated if altered PAK1 expression is important in the type I of the disease.

In conclusion, we utilize several avenues to characterize the PAK1 expression profile in the tumor progression of GEJ adenocarcinoma. Overexpressed PAK1 is a molecular signature of GEJ tumorigenic progression. Upregulation of PAK1 is associated with malignant properties mainly relevant to invasion and metastasis and correlates with poor overall survival. PAK1 overexpression may serve as an independent high-risk prognostic predictor that holds therapeutic promise for GEJ adenocarcinoma.

## Supporting Information

Figure S1 **Correlation between PAK1 and HER-2 expression.** Representative photos of PAK1 (A&B) and HER-2 (C&D) protein expression in GEJ adenocarcinoma from the same patient. Scale bars =  50 µm. Scale bars =  50 µm. (E) The PAK1 protein levels were positively correlated with the HER-2 protein levels in GEJ adenocarcinoma (Pearson's correlation coefficient test, r = 0.366 and *P* = 0.012). (F) The PAK1 transcript levels were positively correlated with the HER-2 transcript levels in GEJ adenocarcinoma (Pearson's correlation coefficient test, r = 0.423 and *P* = 0.022).
(TIF)

Figure S2 **Representative photos presenting PAK1 protein expression in tumors of different histological grades.** (A) high histological grade. (B) middle histological grade. (C) low histological grade. Scale bars =  50 µm.
(TIF)

Figure S3 **Correlation between PAK1 and PCNA expression.** (A–D) Representative photos of PAK1 and PCNA protein expression in GEJ adenocarcinoma. Scale bars = 50 µm. (E) The PAK1 protein levels were not positively correlated with the PCNA index in GEJ adenocarcinoma (Pearson's correlation coefficient test, r = 0.256 and *P* = 0.086). (F) The PAK1 transcript levels were not positively correlated with the PCNA transcript levels in GEJ adenocarcinoma (Pearson's correlation coefficient test, r = 0.348 and *P* = 0.065).
(TIF)

Figure S4 **Representative photos presenting PAK1 protein expression in tumors with varying extents of cancer–cell infiltration into surrounding tissues.** Strong PAK1 protein expression in tumors with more severe cancer–cell infiltration into surrounding tissues (A&C) as compared with faint PAK1 protein expression in tumors with less severe cancer–cell infiltration into surrounding tissues (B&D). (A–B) PAK1 protein expression. (C–D) HE staining. Scale bars = 50 µm.
(TIF)

Table S1 **PAK1 in Primary tumors and paired nontumors were evaluated by immunoblot**
(DOC)
