# Supplementary figures and images for: Prognostic Importance and Therapeutic Implications of PAK1, a Drugable Protein Kinase, in Gastroesophageal Junction Adenocarcinoma
Source: PLoS One. 2013 Nov 13;8(11):e80665. doi: 10.1371/journal.pone.0080665 (PMC3827444; doi:10.1371/journal.pone.0080665)

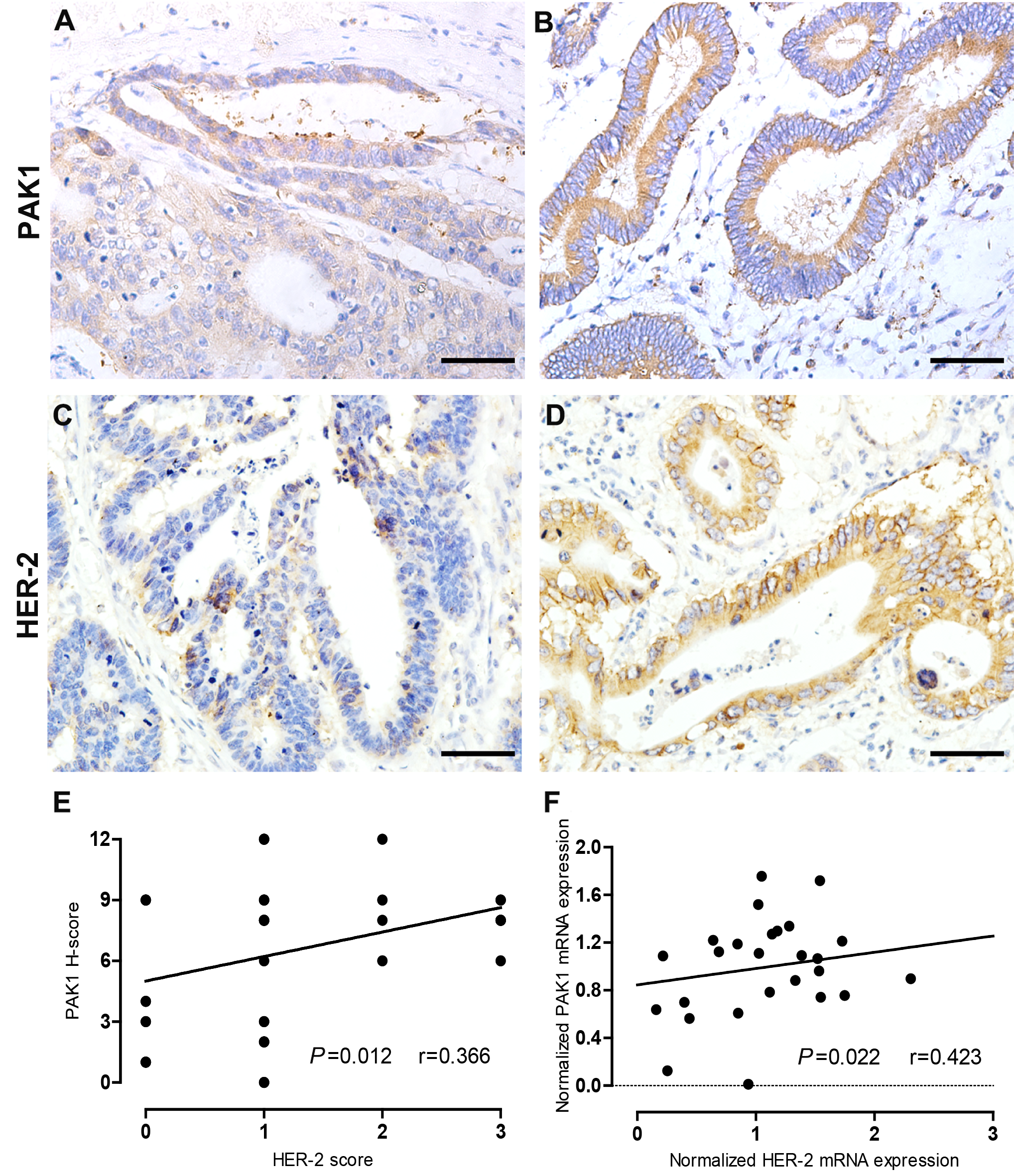

Supplement: Figure S1 — Correlation between PAK1 and HER-2 expression. Representative photos of PAK1 (A&B) and HER-2 (C&D) protein expression in GEJ adenocarcinoma from the same patient. Scale bars = 50 µm. Scale bars = 50 µm. (E) The PAK1 protein levels were positively correlated with the HER-2 protein levels in GEJ adenocarcinoma (Pearson's correlation coefficient test, r = 0.366 and P = 0.012). (F) The PAK1 transcript levels were positively correlated with the HER-2 transcript levels in GEJ adenocarcinoma (Pearson's correlation coefficient test, r = 0.423 and P = 0.022). (TIF) [file pone.0080665.s001.tif]

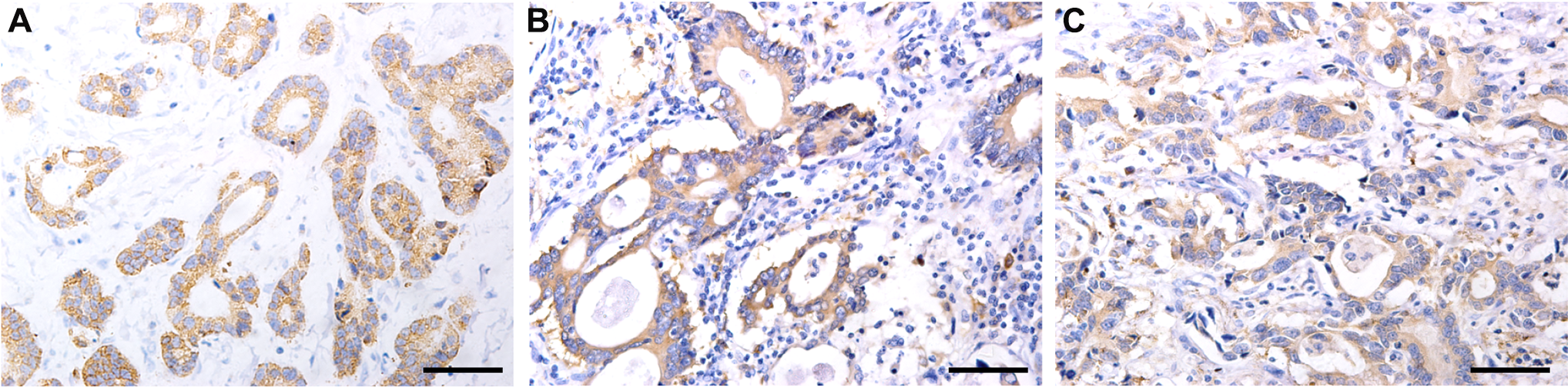

Supplement: Figure S2 — Representative photos presenting PAK1 protein expression in tumors of different histological grades. (A) high histological grade. (B) middle histological grade. (C) low histological grade. Scale bars = 50 µm. (TIF) [file pone.0080665.s002.tif]

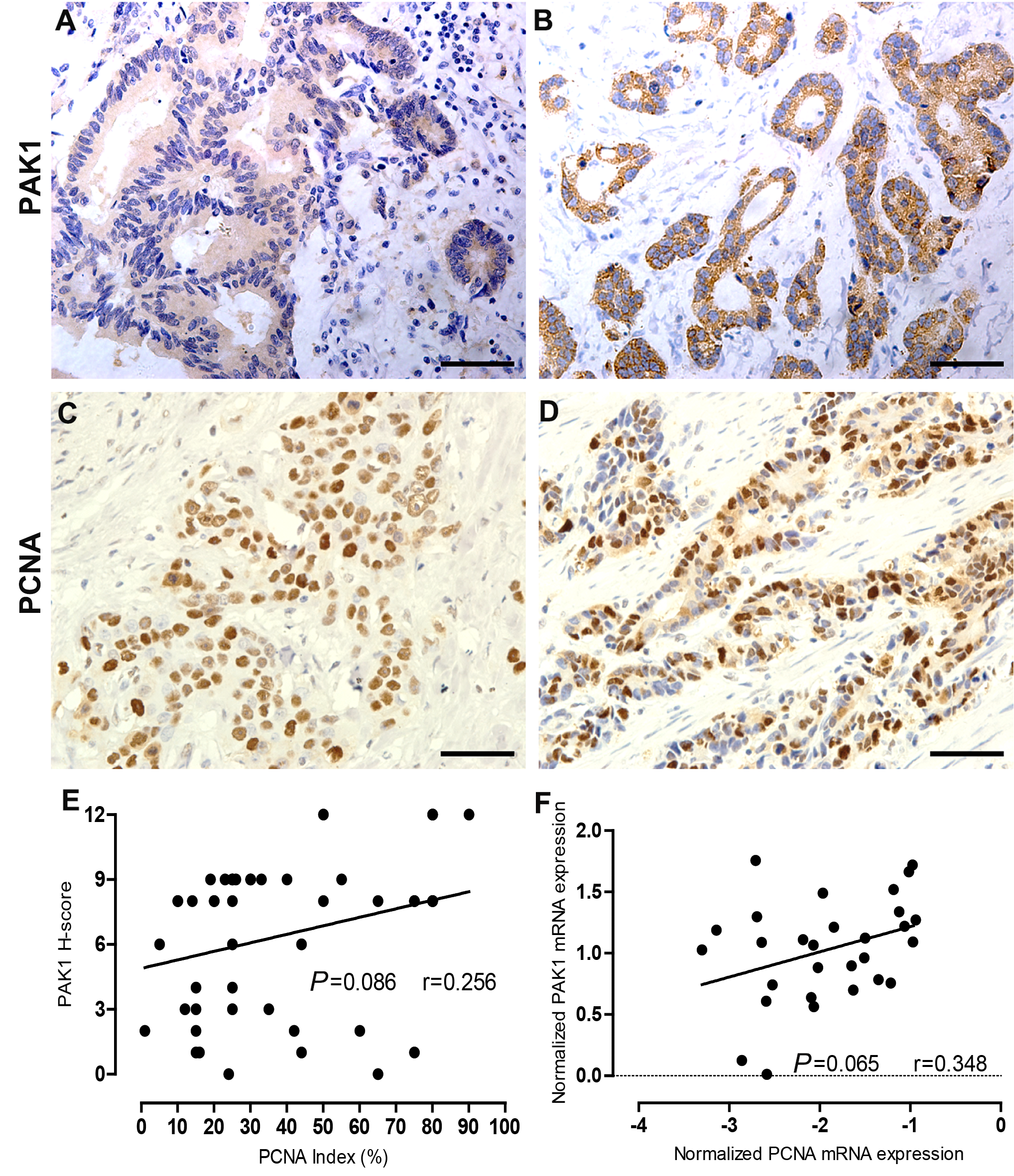

Supplement: Figure S3 — Correlation between PAK1 and PCNA expression. (A–D) Representative photos of PAK1 and PCNA protein expression in GEJ adenocarcinoma. Scale bars = 50 µm. (E) The PAK1 protein levels were not positively correlated with the PCNA index in GEJ adenocarcinoma (Pearson's correlation coefficient test, r = 0.256 and P = 0.086). (F) The PAK1 transcript levels were not positively correlated with the PCNA transcript levels in GEJ adenocarcinoma (Pearson's correlation coefficient test, r = 0.348 and P = 0.065). (TIF) [file pone.0080665.s003.tif]

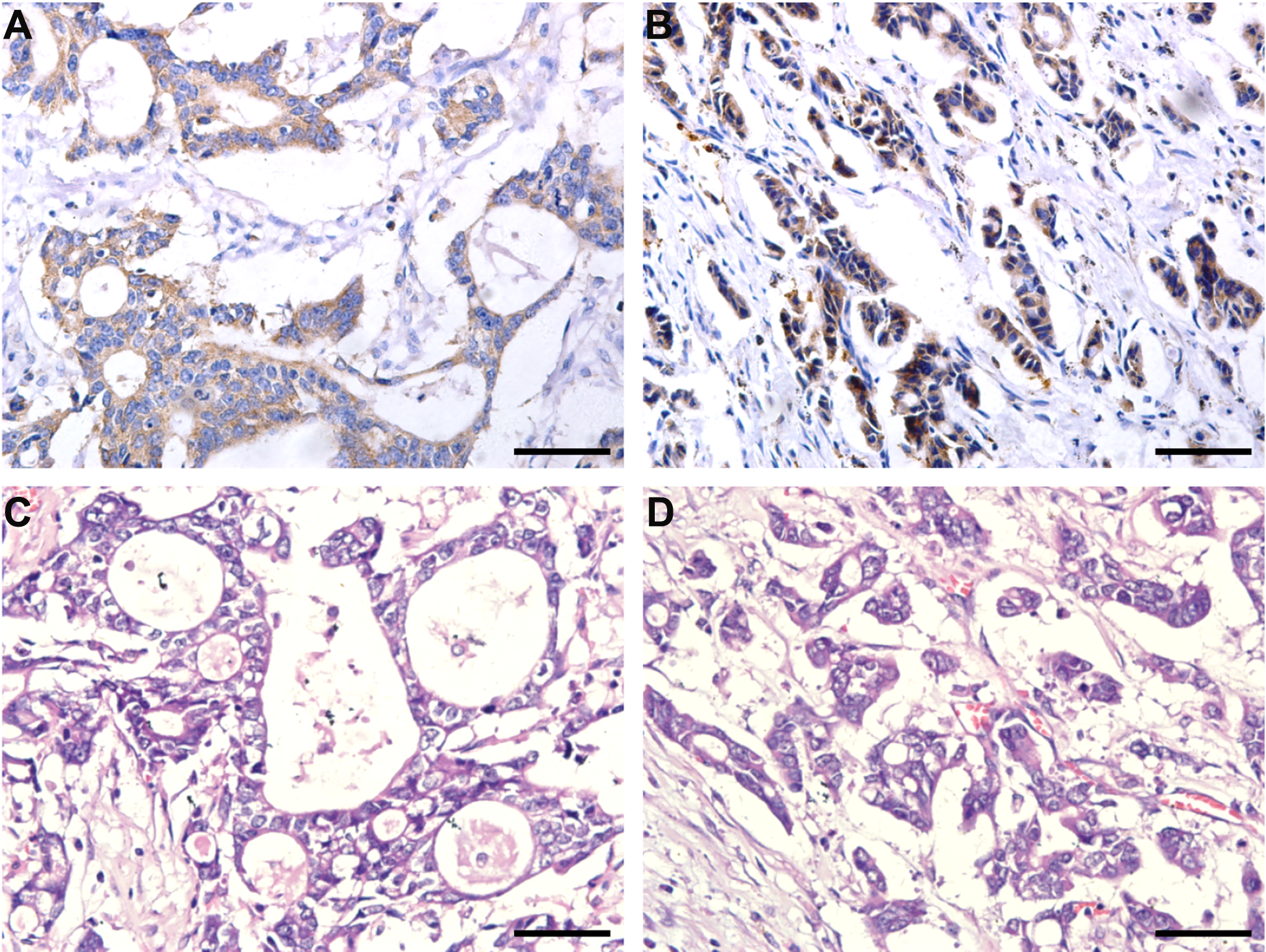

Supplement: Figure S4 — Representative photos presenting PAK1 protein expression in tumors with varying extents of cancer–cell infiltration into surrounding tissues. Strong PAK1 protein expression in tumors with more severe cancer–cell infiltration into surrounding tissues (A&C) as compared with faint PAK1 protein expression in tumors with less severe cancer–cell infiltration into surrounding tissues (B&D). (A–B) PAK1 protein expression. (C–D) HE staining. Scale bars = 50 µm. (TIF) [file pone.0080665.s004.tif]
