# Supplementary material for: Prognostic Importance and Therapeutic Implications of PAK1, a Drugable Protein Kinase, in Gastroesophageal Junction Adenocarcinoma
Source: PLoS One. 2013 Nov 13;8(11):e80665. doi: 10.1371/journal.pone.0080665 (PMC3827444; doi:10.1371/journal.pone.0080665)
Supplement: Table S1 — PAK1 in Primary tumors and paired nontumors were evaluated by immunoblot (DOC) [file pone.0080665.s005.doc]

**Tables**

| **Table S1. PAK1 in Primary tumors and paired nontumors were evaluated by immunoblot** | | | | | | | | |
| --- | --- | --- | --- | --- | --- | --- | --- | --- |
| No. | PAK1(T/NT) | Age | Sex | pT | pN | pM | Histological grade | pTNM Stage |
| 1 | 3.691 | 62 | M | 4b | 3 | 0 | 3 | IIIC |
| 2 | 4.391 | 55 | M | 4a | 2 | 0 | 3 | IIIC |
| 3 | 2.697 | 62 | W | 4a | 0 | 0 | 3 | IIB |
| 4 | 38.152 | 61 | M | 4a | 0 | 0 | 3 | IIB |
| 5 | 102.538 | 59 | M | 4 | 3 | 0 | 2 | IIIC |
| 6 | 1.838 | 65 | M | 1 | 0 | 0 | 1 | IA |
| 7 | 6.325 | 70 | M | 3 | 0 | 0 | 2 | IIA |
| 8 | 0.856 | 58 | M | 2 | 0 | 0 | 2 | IB |
| 9 | 42.643 | 73 | W | - | - | 1 | - | no surgery |
| 10 | 391.954 | 61 | W | 4a | 0 | 0 | 2 | IIB |
| 11 | 1.253 | 57 | M | 4a | 1 | 0 | 2 | IIIA |
| 12 | 66.329 | 63 | M | 4a | 1 | 0 | 2 | IIA |
| 13 | 1.848 | 66 | M | 2 | 0 | 0 | 2 | IB |
| 14 | 2.252 | 51 | M | 4a | 2 | 0 | 3 | IIIB |
| 15 | 5.187 | 50 | M | 4a | 1 | 0 | 2 | IIIC |
| 16 | 58.603 | 67 | M | 4a | 1 | 0 | 3 | IIIB |
| 17 | 2.375 | 48 | W | 4a | 1 | 0 | 3 | IIIA |
| 18 | 1.096 | 65 | M | 4a | 1 | 0 | 3 | IIIA |
| 19 | 16.190 | 61 | M | 3 | 3 | 0 | 2 | IIIC |
| 20 | 2.450 | 72 | M | 3 | 1 | 0 | 2 | IIIA |
| pT, depth of invasion; pN, degree of spread to regional lymph nodes; pM, presence of distant metastasis | | | | | | | | |
|
